# Factors influencing mode of transport in older adolescents: a qualitative study

**DOI:** 10.1186/1471-2458-13-323

**Published:** 2013-04-10

**Authors:** Dorien Simons, Peter Clarys, Ilse De Bourdeaudhuij, Bas de Geus, Corneel Vandelanotte, Benedicte Deforche

**Affiliations:** 1 Department of Human Biometry and Biomechanics, Faculty of Physical Education and Physical Therapy, Vrije Universiteit Brussel, Pleinlaan 2, Brussels, B, 1050, Belgium; 2 Department of Movement and Sport Sciences, Faculty of Medicine and Health Sciences, Ghent University, Watersportlaan 2, Ghent, B, 9000, Belgium; 3 Fund for Scientific Research Flanders Belgium, Egmontstraat 5, Brussels, B, 1000, Belgium; 4 Department of Human Physiology and Sportsmedicine, Faculty of Physical Education and Physical Therapy, Vrije Universiteit Brussel, Pleinlaan 2, Brussels, B, 1050, Belgium; 5 Institute for Health and Social Science Research, Centre for Physical Activity Studies, Central Queensland University, Bruce Highway, Rockhampton, QLD 4702, Australia

**Keywords:** Active transport, Motorized transport, Youth, Focus groups, Transport choice

## Abstract

**Background:**

Since a decline in activity levels occurs in adolescence, active transport could be important to increase daily physical activity in older adolescents (17–18 years). To promote active transport, it is necessary to be aware of the barriers and facilitators of this type of transport, but also of other transport modes. This study sought to uncover the factors influencing the choice of transport mode for short distance travel to various destinations in older adolescents using focus groups.

**Methods:**

Thirty-two focus group volunteers (mean age of 17 ± 1.2 years) were recruited from the two final years of the secondary school in Antwerp (Belgium). Five focus groups were conducted (five to eight participants/group). Content analysis was performed using NVivo 9 software (QSR International). Grounded theory was used to derive categories and subcategories.

**Results:**

Data were categorized in three main themes with several subcategories: personal factors (high autonomy, low costs and health), social factors (good social support) and physical environmental factors (short travel time, good access to transport modes and to facilities, good weather, an adapted built environment, perceived safety and ecology).

**Conclusions:**

For older adolescents, the interplay between short travel time, high autonomy, good social support, low costs, good access to transport modes and facilities, and good weather was important for choosing active transport over other transport forms for travelling short distances to various destinations. Other well-known factors such as safety, ecology and health seemed not to have a big influence on their transport mode choice.

## Background

Active transport (i.e. walking, cycling,…) is a type of physical activity that offers health benefits to adolescents, such as higher levels of daily physical activity [1], lower odds of being overweight or obese [2,3], higher levels of cardiovascular fitness [4,5] and a better cognitive performance [6]. An increase in active transport might also reduce traffic congestion and CO_2_ emissions [7]. Since a steep decline in activity levels and in active transport occurs in adolescence (12–18 years) [1,8,9], it could be important to focus on active transport to increase the daily physical activity in older adolescents (17–18 years) [10]. Especially since Belgian adolescents are allowed to drive a moped from the age of 16 and a car from the age of 18. Older adolescents also become more independent, they perceive less parental control and more peer pressure [11] and they are allowed to purchase and consume alcohol from the age of 16. Furthermore, as physical activity tracks stronger from late adolescence to adulthood than from early adolescence to adulthood [12,13], increasing walking or cycling for transport in older adolescents may be particularly important because this transport choice may persist into adulthood.

Designing effective interventions to promote active transport in older adolescents requires a comprehensive understanding of the correlates of active transport [14]. Ecological models state that physical activity (including active transport) is influenced by an interplay between psychosocial, sociodemographic and physical environmental factors and each of these factors needs attention in research [15,16]. To date, most research investigating correlates of active transport in adolescents has only focused on young adolescents (12–16 years) [17,18] and on active transport to/from school [15,17-21]. However, correlates of active transport are likely to differ from young adolescence to older adolescence. Furthermore, as the most consistent correlate of active commuting is distance to school [19,20,22,23], it might also be important to promote active transport to other destinations within older adolescents neighborhoods, given that they may not live within easy walking or cycling distance from school. The criterion distance for active transport to school in older adolescents in Belgium could be set at eight kilometers for cycling and two kilometers for walking [20]. But even within the distance of eight kilometers, approximately 40% of adolescents use passive transport to go to school, as shown in a Belgian and a UK study. [20,23]. A review of qualitative studies on the views of children, young people and parents about walking and cycling [24] also described a culture of car use. They found that young people perceive active transport as less safe, pleasant and convenient than car travel. Therefore, it might be important to conduct an in-depth investigation of the factors influencing transport mode choice for short distances (≤ 8 km) in older adolescents. To our knowledge, no studies on active transport in (older) adolescents to destinations other than school have been conducted.

Not only knowledge about correlates of active transport is important, it is also necessary to be aware of the barriers and facilitators of other modes of transportation. Knowing why older adolescents choose to drive a moped or ask their parents for a ride might help in explaining participation in active transport. There is no information on which factors push older adolescents into the use of mopeds for travelling short distances once they reach the age they are allowed to drive these motorized vehicles. Since transport habit is a strong correlate of transport mode choice [25], it is important to promote active transport before the use of motorized transport modes for travelling short distances becomes a habit. Furthermore, public transport might be a good alternative for active transport, as the additional minutes of walking before and after use of public transport may help to increase activity levels and reduce health risks [26,27].

To date, not much is known about the factors influencing transport choice for short distance travel to various destinations in older adolescents. There is a need for qualitative studies to explore this research area, as qualitative research methods offer a broad and in-depth insight into the individuals’ experiences and perceptions [28]. Focus groups provide the possibility to learn and get a broad range of information about topics that are poorly understood (travel choices), especially in particular segments of the population, such as older adolescents [29]. Interactive group discussions stimulate a process of sharing and comparing, and different points of view are revealed [30].

Therefore, a qualitative study was conducted in order to explore the factors influencing the choice of transport mode for short distance travel to various destinations in older adolescents.

## Methods

### Sampling

Focus group participants were recruited in March 2012 from a secondary school in Antwerp (Belgium), and consisted of 32 volunteers from the two final years (mean age of 17 ± 1.2 years) in general, technical, occupational and artistic studies. These are the four main study disciplines available for secondary school students in Belgium, in which general studies prepare for higher education, technical studies have a more technical and practical approach, occupational studies are very job specific and artistic studies combine general education and art practice. Because distance is a very dominant barrier for active transport, the study was conducted in the city of Antwerp. With 506,225 inhabitants and a population density of 2,478 inhabitants/km^2^, Antwerp has a single condensed urbanized built-up area with plenty of destinations for short distance transportation [31]. The study protocol was approved by the ethics committee of the university hospital of the Vrije Universiteit Brussel. All participants agreed to participate in the study via informed consent and gave permission for their quotes to be used in research publications.

### Research protocol and measures

The protocol consisted of two parts: a brief and basic questionnaire followed by focus group discussions. First, the participants completed a questionnaire collecting sociodemographic data, data about transport modes, transportation preferences, distance to school and driver license possession. Physical activity was also assessed with one self-report question. Such single questions have shown to have a good validity in studies among adults where physical activity is not the primary focus and more detailed measures are not feasible [32]. Focus groups were held until saturation (a point at which all questions have been thoroughly explored in detail and no new concepts or themes emerge in subsequent interviews) was reached, since a sample size cannot be pre-determined given the need for a thorough exploration of an as yet unknown behavior (transport mode choice for short distance travel) [33]. In total, five focus groups were held (divided by study discipline), with a range of five to eight participants per group. All focus groups were conducted in Dutch and lasted approximately 50 minutes. A focus group protocol and a semi-structured discussion guide (see Table 1) were developed in consultation with all co-authors and were consistent with recommended focus group methodology [34]. The guide consisted of several questions, including an opening question, an introduction question, a transition question, five key questions and an ending question. Most of the discussion time was spent on the key questions, asking which factors determine adolescents’ transport mode choice to school and to other nearby destinations, whether and why their transport mode choice changed in the last three years and the advantages and disadvantages of the different types of transportation for short distance travel (≤ 8 km). The questions aimed to uncover facilitators and barriers of all types of transportation. The moderator (D.S.) used the focus group guide to lead the discussions, allowing ample time for participants to respond to questions and comments from other participants. In addition, designated observers were present to take notes and to make sure the moderator did not overlook any participants trying to add comments. The students were offered an incentive (movie ticket) for their participation in the focus group discussions. With permission of the participants, all conversations were audio-taped and filmed, to inform the transcription of focus group interviews.

**Table 1:** Semi-structured discussion guide

**Question type**	**Purpose**	**Question**	**Timing**
Opening	*To get participants acquainted and feel connected*	1. Tell us your name, study and whether or not you have a moped -or a driver license.	5 min
Introduction	*To begin discussion of topic*	2. What springs to mind when you hear the term “active transportation”?	5 min
Transition	*To move towards the key questions*	3. A) Which transport mode do you use most often to go to school?	5 min
		→it can also be a combination	
		→same transport mode to and from school?	
		B) Which transport mode do you use to go to other destinations in your neighborhood (short distance)? Such as sport clubs, shops, friends…	
Transition		4. A) What is, according to you, a feasible distance to cycle to destinations?	5 min
		B) From research, it shows that 8 km is feasible. What do you think of that?	
Key		5. Who thinks he lives on a feasible walking or cycling distance from school? Which elements determine your choice of transportation?	10 min
		- weather	
		- physical environment	
		- habit	
		- influence of parents, friends, partner…	
		- fatigue	
		- safety	
		- financial status	
		- time (faster/slower)	
		- health (more active…)	
		- ecological aspect	
		- other intrinsic or extrinsic motivational factors…	
		6. Which elements determine your choice of transportation to other destinations in your neighborhood ? Such as sport clubs, shops, friends…	10 min
Transition		7. Think back at the 3 previous years. Did you always go with the same transport mode to destinations in your neighborhood, or was there a switch in transport mode?	5 min
Key	*To obtain insight about areas central to the study*	8. Think back at the 3 previous years. Which elements made you maintain transport mode or which elements made you switch transport mode to destinations in your neighborhood?	10 min
		- moped –and/or car driver license	
		- physical environment	
		- social influence (parents, friends, partner…)	
		- financial status	
		- safety	
		- health reasons	
		- other intrinsic or extrinsic motivational factors…	
		9. A) According to you, what are the advantages and disadvantages of walking and cycling to destinations?	
		B) According to you, what are the advantages and disadvantages of using a moped, a motorcycle or a car to go to destinations?	10 min
Key		10. How would you encourage adolescents of your age to cycle or walk more to destinations in their neighborhood?	15 min
		11. Which channels/media would you use to encourage adolescents of your age to cycle or walk more to destinations in their neighborhood?	
Ending	*To determine where to place emphasis and to bring closure to the discussion*	12. Choose one element that influences your choice of transportation the most.	5 min
Ending		13. Is there anything that we should have talked about but didn’t?	5 min

### Data analysis

Data obtained by the questionnaire were entered into an SPSS-file (version 20.0) to calculate descriptive statistics. Data from the audio tapes were transcribed verbatim. Transcripts of the focus group conversations were entered into NVivo 9 qualitative software (QRS International) to analyze the data, based on grounded theory. Grounded theory is a method of analyzing qualitative data which is grounded in the data without preconceived theories and is characterized by intensively analyzing data, often sentence by sentence, or phrase by phrase [35]. Codes were developed by DS throughout the focus groups and during the transcription of the audio recordings according to the responses and the themes which arose frequently and were relevant to the aim of the study. DS assigned segments of the transcripts to the codes (segments could be assigned to multiple codes). Codes were then grouped into broader categories. The codes to be used and the assignment of segments to codes were validated by two other researchers (JVC, TD). Doubts or disagreements were discussed until consensus was reached.

As suggested by Sandelowski [36] and previously used by Van Cauwenberg et al. [37], the qualitative data are reinforced by quantitative counts of the participants discussing certain factors influencing their choice of transportation. Thus, when a factor was discussed by less than 25%, we called it “few”, for between 25% and 50%, we called it “some”, for between 50% and 75%, we called it “a lot of” and for more than 75% of the participants, we called it “almost all” in the results’ description.

## Results

### Descriptives

Descriptive statistics are described in Table 2. There were slightly more boys (65.5%) attending the focus groups than girls. Adolescents reported less cycling to school (59.4%) compared to other destinations (81.3%), and a greater use of car/moped (12.5%) and public transport (28.1%) to school than to other destinations (respectively 3.1% and 15.6%). No adolescents in this study owned a car driver license.

**Table 2:** Demographics and transportation and physical activity behavior

**Demographics**	
Age (years) (M ± SD)	17 ± 1.2
% male	65.6
% general study	43.8
% technical/occupational/artistic study	56.3
% living in/at the edge of the city	75.0
% driver license moped	12.5
**Transportation and physical activity behavior**	
% cycling/walking to school	59.4
% car passenger to school	3.1
% moped to school	9.4
% public transport to school	28.1
% mostly walking/cycling to other destinations	81.3
% mostly car/moped to other destinations	3.1
% mostly public transport to other destinations	15.6
% walking/cycling as favorite transport mode	78.1
% car/moped as favorite transport mode	9.4
% public transport as favorite transport mode	12.5
% moderately physically active	37.5

### Content analysis

Qualitative data analysis revealed three main themes with several factors that affected choice of transport mode in older adolescents: personal factors (including autonomy, financial aspect and health), social factors (including social influence) and physical environmental factors (including travel time, accessibility (access to transport modes and access to facilities), weather, built environment, perceived safety (traffic safety and safety from crime) and ecology).

#### Personal factors

##### Autonomy

A lot of participants said that they do not like to be dependent on something or someone when it comes to transportation. This was considered an advantage of active transport. Cycling to destinations provides the adolescents with a great deal of independence because it is very reliable, they can go and leave when they want, even in the evening or at night, it provides them with a direct route to their destination and they are independent from their parents driving them. Only walking offers an even greater amount of freedom, as mentioned by few participants, because then you do not have to think about the keys or lights for the bicycle. But this was only considered practical for very short distances. On the other hand, public transport has a lot of disadvantages that impacted negatively on autonomy, such as long waiting times, delays, traffic jams, limited availability in the evening and only access to a limited amount of destinations. For example, one boy said: *“If you go somewhere by bicycle, you have all the freedom. If you want to change direction on the go or something like that, you do not have to wait half an hour at the bus stop to take another bus. And you can immediately do what you want.”* Some adolescents do think that a car would provide them with even more freedom and independence, especially for long distances. But for short distances in the city a car would be less convenient than cycling or walking due to traffic congestion, one way streets and a lack of parking spots.

#### Financial aspect

Some adolescents talked about the financial aspect of transportation. The main reasons for not getting a moped license and buying a moped were the costs. A female participant said: *“I wouldn’t buy a moped because you can only drive it from the age of 16 and if you wait 2 years, you can have a driver license and a car is much faster. Why spend extra money if it isn’t necessary?”*

Cost was also considered a barrier for using public transport by some participants. They said that it is rather expensive when you have to buy a ticket every time. When their parents would pay for the ticket or when they had a subscription (also paid for by the parents) they would take public transport more easily. This was illustrated by a male participant saying: *“I don’t have a subscription so if I want to take the bus I have to consider whether I still have any money. Otherwise, I’ll have to walk or cycle.”*

#### Health

Few participants mentioned physical health as a factor in the choice of transport mode. Some of them thought it is just an extra benefit that riding a bicycle is good for their physical fitness, and others even doubted whether cycling in a city is healthy. They suggested that the car exhausts have a rather negative influence on their health. A female participant mentioned: *“My mom says I have to cycle because it’s healthy, but I don’t think it is very healthy. All those car exhaust you’re inhaling counter the health benefits of cycling a distance.”*

### Social factors

#### Social influence

It was mentioned by a lot of adolescents that friends, parents and partners have an influence on their transport mode. That influence can be positive or negative. For instance, a lot of adolescents like to cycle to destinations with one or more friends. They would also cycle longer distances when they are not alone. But if the friends in their environment regularly take public transport, have a drivers or a moped license, they will also join in these motorized transport modes. This was illustrated by a male participant who said: *“If I go out with friends and they would go by car it is logical that I will also ride along. So you do not have to go alone. It depends on what they choose, those friends, and then you automatically go along.”* Adolescents with a moped mentioned that the social aspect was one of the most important factors in driving a moped.

When meeting friends at the destination, a few adolescents mentioned they do not like to arrive sweaty and red-faced, so they might consider not taking a bicycle for this reason. Few participants said that their parents encouraged them to use active transport by setting example or by not allowing a ride with the car. However, few others also said that their parents like to drive them to destinations by car when the weather is bad or because they do not want to use active transport themselves. Adolescents with partners with a moped –or driver license, also mentioned often getting a ride from their partner.

### Physical environmental factors

#### Travel time

A lot of participants stated that, to travel short distances, they usually choose the fastest transport mode. In the city this is mostly the bicycle. For example, one female participant said: *“Yes, if you go by bicycle, you are always faster than going by bus. Because by bicycle, you can choose your own pace and you don’t have to wait as long as for public transport*”. Adolescents with a moped license also mentioned travel time as a facilitator of driving a moped. With a moped, they have the advantages of cycling, but travel time is even shorter. When they have sufficient time, some adolescents might choose public transport or walking.

#### Accessibility

##### Access to transport modes

Access to transport modes was discussed by some participants. None of the adolescents had a driver’s license yet, so access to a car was limited to being a passenger. Some of them said that, sometimes they could ride as a passenger with parents or friends and few also admitted that if they would have their driver’s license and a car, they would probably often go by car. Adolescents who regularly took public transport, said that this was because public transport stops were near to their home. For example, one boy said: *“I do not live far from the transit zone for all the busses and that is an advantage for me. I have all the options. I can take any bus to school or back home. Public transport is always the first choice for me.”* If a public transport stop is not nearby, the adolescents mostly cycle or walk to their destination. Almost all adolescents mentioned having a bicycle, except for few whose bicycle got stolen. Only few adolescents mentioned having a moped.

##### Access to facilities

Some participants mentioned that it is important to have access to good bicycle storage or a bicycle parking at their destination. If not, they would consider taking an alternative transport mode because they do not want their bicycle or moped to get stolen.

Some adolescents considered bicycle sharing programs as very practical transport modes. One participant indicated: *“I like the bicycles that are spread all over the city and that you can rent for a very cheap price. Because there are so many of those ‘bike-stations’ in the city, it gives an easy access to transportation.”* When their own bicycle is not available or broken and there is no good access to public transport, some adolescents mentioned using these shared bicycles.

#### Weather

The weather is an important factor in choosing a transport mode, according to a lot of adolescents. In rain, snow or ice, they like to take motorized transportation such as public transport or a ride with a car. Some of them also admitted that, in bad weather, they would probably go by car once they have their driver license. Although few of them stated that, with the appropriate rain gear, they would still take their bicycle. For example, one female participant said: *“If it’s raining a little bit it is not a problem to cycle but if it’s pouring rain I would ask my mom to give me a ride.”*

#### Built environment

Some participants mentioned the built environment as a factor in choosing between transport modes. A good cycling path and roads that are not too busy are important for choosing to cycle. A female participant indicated: *“For instance, if the road is really bad, with holes and bumps and all, then you have to make a detour. And then you have to cycle for 15 minutes more. Then I would ask my parents to drive me.”* When it comes to cars, few said that, if they had a driver license, they would not take their car to certain destinations in the city because of the lack of parking lots and the traffic jams.

#### Safety

Although a lot of participants said something about safety, it did not seem to be a very important factor of transport mode choice. They reported that it is mostly a matter of being careful yourself and that they would not change their transport mode for safety reasons.

Traffic safety: Some of the adolescents mentioned traffic safety as a factor influencing their transport mode choice. They said that, by riding a bicycle, they are vulnerable road users. Busy traffic and a lack of good and clear cycling paths make cycling more dangerous, but according to the adolescents, these aspects do not make them change transport mode. On the other hand, snow and ice on cycling paths does make some adolescents switch to motorized transportation. Although none of them had a driver’s license for motorcycles, they were considered very dangerous by the participants. For example, one girl said: *“My mother has a motorcycle and she already fell a couple of times. And my stepfather also has a motorcycle and he was even hospitalized once. So I do not want to do that. I do not want a motorcycle, no way.”*

Safety from crime: Personal safety was only brought up by a few adolescents. They mentioned that they did not want to cycle to certain places because they are afraid their bicycle might get stolen.

#### Ecology

Only few participants said something about ecological aspects. They thought that it is a disadvantage that a car is bad for the environment and an advantage that cycling and walking is good for the environment, but that this would not be a decisive factor in transport mode choice. For instance, a female participant said: *“It’s not because you suddenly start cycling that the environment is going to get better. There’s no point in cycling for that reason.”*

## Discussion

The current study used focus groups to investigate the influencing factors of transport mode choice for short distance travel to various destinations in older adolescents. The findings show that choosing between transport modes is not influenced by one factor, but by a combination of factors which influence each other. For older adolescents, the interplay between short travel time, high autonomy, social factors, low costs, good access to transport modes and facilities, and good weather were the most important factors in favor for choosing active transport over other transport forms. Other well-known factors such as safety, ecology and health do not seem to have a big influence. These factors may not be as important focus points when making policy to promote active transport in older adolescents.

In this study, cycling was the most popular transport mode. Throughout the focus group conversations, it became clear that a bicycle offers several benefits that are particularly salient for older adolescents. First of all, cycling is a fast way of travelling in urban areas. Secondly, it offers them autonomy. This aspect is not yet discussed in research, most likely because in previous research focus has been on younger adolescents [17,18], and they receive lesser freedom from their parents to travel independently in comparison to older adolescents. For older adolescents, it is important that their transport modes are flexible. With a bicycle, it is possible to go to all (nearby) destinations at all times, whenever they want and at their own pace. Thirdly, the social aspect of cycling is very appealing. Older adolescents like cycling together with friends and might also cycle longer distances when they are not alone. This is in line with previous research in youth (5–18 years), older adolescents (17–18 years) and adults, where social support and modeling was positively associated with cycling for transport [20,38-40].

The popularity of cycling could be explained by the fact that this study was conducted in Flanders, Belgium. Because of geographical and climatological advantages (flat landscape, many urban areas, short distances, not too warm…), Flanders has a real ‘cycling mentality’ with 34.7% of the households owning 3 or more bicycles and 26.2% of the population cycling at least once a week for transportation [41]. Previous research conducted in Flanders also found a high percentage of cycling in older adolescents [20]. This is also reflected in bicycle sharing programs (the shared use of a bicycle fleet), which is relatively new, but very popular in Belgium. The adolescents find these bicycles cheap, practical and very accessible. Although bicycle sharing offers several social and environmental benefits and has become increasingly popular across the globe [42], research concerning this type of transport is still scarce.

The main barrier for cycling in this study, is the weather. This is in line with the study of Yang et al. [43], who stated that the use of active transport in adolescents exhibited clear seasonal patterns: high during summer months and low during winter months. Winters et al. [44], also found that fewer people cycled in cities with more days of precipitation or freezing temperatures. Although weather is a factor that cannot be influenced, the consequences of bad weather are changeable. For instance, in winter, snow or ice on walking and cycling paths should be removed, making it safe and more pleasant to use active transport.

Safety, which is regularly stated to be a barrier of active transport in previous research in children and young adults [24], was less important for older adolescents’ choice of transport in the present study. This is in line with a study among adolescents of Forman et al. [45] and could be explained by the age-group, as older adolescents get to make more travel decisions themselves and are less influenced by parental concerns about safety issues than younger adolescents [46]. A second explanation could be the ‘safety in numbers’ phenomenon [47], which says that the average cyclist is safer in communities where there is more bicycling because motorists adjust their behavior in the expectation of encountering cyclists. On the other hand, concerns about bicycle theft were a barrier for cycling in this study. As suggested by the adolescents themselves, more bicycle storage and safe bicycle parking would be required to promoting active transport. Research on bicycle theft and bicycle storage is scarce, although a study of Titze et al. [48] found that safety from bicycle theft was positively associated with regular cycling in a population of university students.

Walking as a transport mode has several advantages in line with those from cycling. But as the older adolescents want their travel time to be as short as possible, walking is probably more suitable as transport mode for people in other age categories, such as older adults [49]. Although in a study on active transport and physical activity in adolescents in ten European countries commuting by bicycle was reported less frequently than walking [1].

In this study, only a few adolescents drove mopeds. This is in line with the results from the Travel Behavior Research Flanders, who found that only 2.27% of older adolescents in Flanders use a moped as their main transport mode [41]. The main barrier for not driving a moped is the financial aspect. Facilitators for driving mopeds were the short travel time and the social aspect. To the best of our knowledge, no previous research on barriers or facilitators of moped use in older adolescents has been conducted.

Results showed that public transport is not very popular in older adolescents. The public transport system in Flanders has several weaknesses that are especially barriers for older adolescents. For instance, the limited evening, night and weekend schedules make it difficult to participate in social events and nightlife activities using public transport. This finding is consistent with the results of Calafat et al. [50], who found that the provision of late night public transport is essential for young individuals in the prevention of traffic risk behaviors during nightlife. The frequent delays and long waiting times between vehicles are also negative since a short travel time is important for older adolescents. Furthermore, older adolescents find public transport rather expensive. Indeed, a study of Jones et al. [51], where bus fare exemptions for adolescents in London have been studied, showed that eliminating the cost of getting on a bus might positively affect wellbeing in general. It broadens the capacity for all young people to travel independently of adult supervision and opens up a network of public, mobile places in which young people can actively maintain their community of friends in a relatively accessible setting [51]. It might be interesting to investigate how the public transport system can be improved to meet the needs of young people.

Belgian adolescents are allowed to drive a car and a motorcycle from the age of 18. Most adolescents in this study were planning on doing this and were looking forward to driving a car. A car would offer an even greater amount of autonomy. It would also reduce travel time (in case of no traffic jams) and eliminate the barrier of weather. A recent study from Line et al. [52] confirms these findings, as they found that young people often have a strong preference for the car due to the speed, freedom and positive image they believe it would provide them. Driving a motorcycle did not seem to be something adolescents of the present study want to do in the near future. The possible safety issues are a barrier to this transport mode. Previous research did indeed confirm that in Belgium, young adults (19–27 years) are, compared to other age groups, most likely to be involved in a causality accident with a motorcycle [53]. According to Rutter and Quine [54], a particular pattern of behavior, notably a willingness to break the law and violate the rules of safe riding, is associated with motorcycle accidents in youth.

There are several limitations in the present study. First, 75% of the participants lived in or at the edge of the city. So results may not be generalizable to rural areas. Second, active transport was rather high in this group of participants, making it harder to gather why some older adolescents do not use active transport for short distance travelling. Third, results might be different in other countries since Belgium, and specifically Flanders, has good geographical and climatological conditions for cycling and a real ‘cycling mentality’. Fourth, although the interactive aspect of the focus groups is a benefit, it might also cause a social desirability bias. But as this is the first study investigating transport mode choice for short distance travel to various destinations in older adolescents using qualitative data, results are unique, resulting in detailed and in-depth information.

## Conclusions

In summary, for older adolescents, choosing between transport modes for travelling short distances to various destinations is not influenced by one factor, but by a combination of factors which influence each other. Since driving a car is not yet an option, cycling has the most advantages for older adolescents. It is a fast transport mode, it offers a lot of freedom to go to many places at all times and they can easily cycle together with friends. No bicycle storage at the destination and snow and ice on cycling paths are barriers for cycling. On the other hand, walking is only practical for very short distances. Driving a moped also offers advantages such as a fast travel time, autonomy and the social aspect, but the financial costs are a serious barrier. Furthermore, public transport has a lot of disadvantages such as a long travel time and little freedom and flexibility. Health benefits and the ecological aspect are no important factors in choosing between transport modes for older adolescents. If quantitative studies in a representative sample of older adolescents can confirm these findings, researchers should take these factors into account when developing interventions to enhance active transport over short distances to various destinations in older adolescents.

## Competing interests

The authors declare that they have no competing interests.

## Authors’ contributions

DS, PC, IDB and BD developed the study design. DS conducted the data collection. DS performed the data analysis. DS drafted the manuscript and all other authors critically reviewed and revised versions of the manuscript. All authors read and approved the final manuscript.

## Pre-publication history

The pre-publication history for this paper can be accessed here:

http://www.biomedcentral.com/1471-2458/13/323/prepub
